# Demand, capacity, and access of the outpatient clinic: A framework for analysis and improvement

**DOI:** 10.1111/jep.12926

**Published:** 2018-04-17

**Authors:** Erik Martijn van Bussel, Marc Boudewijn Victor Rouppe van der Voort, Ronald N. Wessel, Godefridus G. van Merode

**Affiliations:** ^1^ University Medical Center Utrecht Utrecht The Netherlands; ^2^ St. Antonius Hospital Utrecht Utrecht The Netherlands; ^3^ St. Antonius Hospital Nieuwegein Nieuwegein The Netherlands; ^4^ Maastricht University Medical Center Maastricht The Netherlands

**Keywords:** access time, consultations, hospital outpatient clinics, orthopaedics, supply, waiting

## Abstract

**Rationale:**

While theoretical frameworks for optimization of the outpatient processes are abundant, practical step‐by‐step analyses to give leads for improvement, to forecast capacity, and to support decision making are sparse.

**Aims and objectives:**

This article demonstrates how to evaluate and optimize the triad of demand, (future) capacity, and access time of the outpatient clinic using a structured six‐step method.

**Methods:**

All individual logistical patient data of an orthopaedic outpatient clinic of one complete year were analysed using a 6‐step method to evaluate demand, supply, and access time. Trends in the data were retrospectively analysed and evaluated for potential improvements. A model for decision making was tested. Both the analysis of the method and actual results were considered as main outcomes.

**Results:**

More than 25 000 appointments were analysed. The 6‐step method showed to be sufficient to result in valuable insights and leads for improvement. While the overall match between demand and capacity was considered adequate, the variability in capacity was much higher than in demand, thereby leading to delays in access time. Holidays and subsequent weeks showed to be of great influence for demand, capacity, and access time. Using the six‐step method, several unfavourable characteristics of the outpatient clinic were revealed and a better match between demand, supply, and access time could have been reached with only minor adjustments. Last, a clinic specific prediction and decision model for demand and capacity was made using the 6‐step method.

**Conclusions:**

The 6‐step analysis can successfully be applied to redesign and improve the outpatient health care process. The results of the analysis showed that national holidays and variability in demand and capacity have a big influence on the outpatient clinic. Using the 6‐step method, practical improvements in outpatient logistics were easily found and leads for future decision making were contrived.

## 1 INTRODUCTION

Accessibility to care providers is one of the main challenges that every specialist outpatient clinic has to deal with. Delays in access time are nowadays such a common phenomenon that it seems to be considered as an inevitable burden by the public.^1^, ^2^, ^3^ This acceptation could be a dangerous development; delays in access time are not only unpleasant but also increase the burden to sick or discomforted people and could even become dangerous in the case of (unnoticed) medical urgencies.^4^ This makes accessibility a vital aspect of health care in the pursuit of delivering the best clinical care possible.^5^

From a logistical point of view, waiting is sometimes considered as unavoidable due to the constraints on capacity.^6^ However, seen from other perspectives, the inevitableness of delays in access time is not always shared. A common and frequently used perspective is the regular system perspective of access time.^7^, ^8^ In this perspective, delays are explained as a mismatch between supply and demand.^9^, ^10^ Supply—or capacity leading to the supply—can be seen as the specialist delivering care, while demand is the number of patients requesting for an appointment. This includes demand that is generated by the specialist scheduling future follow‐up appointments. In the most optimal scenario, demand and supply are always synchronized with each other so that no delay in access time nor waste of capacity exists. Unfortunately, demand and supply have always a certain degree of variability and stochasticity. This means that even if they are on average equal but without synchronicity, waiting and utilization will be observed.^9^, ^11^

With this perspective in mind, numerous publications have been written that have studied the possibilities to improve matching demand of and supply for outpatient specialist clinics.^7^, ^10^, ^12^, ^13^, ^14^, ^15^, ^16^, ^17^, ^18^ A significant number of publications focus on reducing the number of no‐shows.^12^, ^19^, ^20^, ^21^ Others try to reduce access or waiting times by dynamic scheduling techniques or computer simulation to predict the required capacity.^17^, ^18^ However, prediction and decision models for outpatient logistics that are usable in daily practice are lacking.

In the logistical struggle of daily outpatient practice, policy makers sometimes try to “solve” delays in access time by manipulating demand and supply, such as discouraging demand for new consultations or stretching up the stake of available capacity by forcing health providers to treat more patients with the same resources. Nonetheless, these measures do not offer a sustainable solution.^22^

To be able to overcome long delays in access time, there is a trend in the literature to focus on redesigning outpatient clinics in order to improve the performance in terms of waiting and capacity utilization. This trend started probably with the model of “advanced access” as first described by general practitioner Mark Murray in 2000.^7^, ^9^, ^10^, ^23^, ^24^ In this redesigned system, the outpatient logistics works with “pull” logistics as an alternative for “push” logistics as is common in traditional health care. A push system schedules the availability of appointments in specialist outpatient clinics based on (predicted) demand, while a pull system authorizes the release of appointments based on system status. In other words, instead of planning appointments based on the available—or forecasted—capacity, actual patients demand determine the completion of supply or capacity. In this system, capacity follows demand instead of preplanned capacity leading to potential postponement of demand.

However, studies using the concepts of “push” and “pull” logistics to analyse demand, supply, and access time in the outpatient clinic are very sparse.^7^ This is regrettable, as evidence‐based studies could give outpatient clinics valuable insights to analyse and optimize their own outpatient logistics. Moreover, studies based on daily outpatient practice could support existing theoretical hypotheses and direct future research of “push” and “pull” logistics.

This study was performed to gain insight in the partly untouched dynamics of real outpatient demand, supply, and access time. This was done by analysing individual patient data of daily practice of an orthopaedic outpatient clinic using a 6‐step method. With the goal of a redesigned health care system in mind, the results were used to seek improvements for the match between demand, supply, and access time. In addition, a model was introduced to forecast demand, to schedule capacity, and to support decisions for future outpatient logistics.

The study was performed using data from a large orthopaedic clinic during one complete year. Three questions were formulated before the data were analysed:

- Does the used 6‐step method provide valuable insights of the outpatient clinic?
- If so, how can patient demand, outpatient capacity, and access time be characterized during the analysed year?
- Does the 6‐step method provide enough tools to improve the outpatient dynamics of this clinic and potentially other clinics as well?

The analysis was done using a structured step‐by‐step method. Not only the results of the analysis were considered as a goal of this study but the usability of the method itself was seen as an important outcome as well. By this way, a blueprint was given for other clinics to be able to understand their own triad of demand, supply, and access time.

## 2 METHODS AND MODELS

This study was performed taking the regular system perspective in mind. In this study design, demand for first consultations was considered as an independent parameter. Follow‐up appointments were (partly) influenced by the specialist, since most follow‐up appointments were made on request of the specialist. This influence was analysed and verified in the data. The system of this outpatient clinic was considered mainly as a push system; capacity was largely based on physician availability and scheduling instead of actual demand. Because of comparable access times in surrounding hospitals, a selection bias based on the access time was not expected. Both patient demand and capacity were considered as statistically normally distributed. This distribution was analysed in the results section.

### 2.1 Six‐step model

The same 6‐step analysis was applied to all relevant parameters of demand, capacity, and access times:

- Step 1: Defining and gathering of the raw data
- Step 2: Analysing the data with basic statistics
- Step 3: Identify trends and striking characteristics
- Step 4: Evaluate demand, capacity, and access time
- Step 5: Create a usable model of future demand and capacity for outpatient decision makers
- Step 6: Formulate conclusions and potential improvements

Patient demand, outpatient supply, and access time of all individual patient appointments were gathered during one complete year. Data were extracted from a large orthopaedic outpatient clinic, consisting of one main outpatient locations and several smaller close‐by locations. No major regional or interregional events took place during the year of analysis. Only appointments with an access time ranging from 1 to 30 days were included. Acute consultations in the outpatient clinic formed only a small part of all consultations with less than 1%. Appointments with an access time longer than 30 days were considered as outliers based on the distribution of the access time as described in the results. All available characteristics per appointment were collected and clustered as given in Table 1. The data were manually evaluated for outliers, missing data, and ambiguities.

**Table 1:** Analysed parameters

Personal Information	Dates	Content of Appointment	Other
Personal number	Date of registration	Type of consultation	Location
Reason for consultation	Date of consultations	Duration of consultation	Specialist

For step 2 and 3, analysing the data with basis statistics and identify trends, every parameter was individually plotted and graphed to discover notable trends. Basic statics using Microsoft Excel version 15.0 (2013) were calculated to gain an understanding of the data and to form a reference for the subsequent steps.

During step 4, evaluate demand, capacity, and access time, comparisons for different periods were made by calculating and comparing basic statistics for different periods and making a concise time series analysis. Linear correlations between parameters were also calculated using Pearson correlations coefficients. Trends of steps 2 and 3 were further evaluated and, if possible, strengthened with more calculations and argumentation.

Theoretical frameworks have no value without practical consequences. Therefore, in the fifth step, a blueprint of a prediction and decision model was given for outpatient decision makers to forecast future demand and needed capacity. The studied outpatient clinic was used as an example.

The last sixth step, formulate conclusions and potential improvements, gives an overview of the most important findings and improvement found in the previous steps.

## 3 RESULTS

### 3.1 Step 1: gathering of the raw data

Data were analysed for the year 2015 from week 2 until week 49. The first and last week (53) were excluded since they fell only partly in 2015. Weeks 50 to 52 were excluded because of incomplete data administration as the result of software updates. For follow‐up consultations, weeks 48 and 49 were excluded as well because of inadequate registration in one of the outpatient clinics. For access time, the same period was used for further analysis.

### 3.2 Step 2: analysing the data using basic statistics

Overall, 56.3% of the patients were seen at the main outpatient location, while the other 43.7% were seen at on the 3 smaller outpatient locations. On average, 38 morning and/or afternoon consultation shifts in the outpatient were completed per week, with a standard deviation of 7.5. A total of 610 no‐shows were registered, consisting of 482 follow‐up appointments and 128 new appointments and making up, respectively, 3.1% and 1.2% of the total number of appointments.

#### 3.2.1 New consultations

A total of 10 474 requests for new consultations were registered. A total of 10 306 new consultations were seen during the same period. The small difference was explained by the access time, leading to a slightly different population of patients requesting for a consultation and patients seen during the same period. The average time between the request for a consultation and the actual appointment was 9.8 days.

The average number of weekly requests for new consultations was 218, with a standard deviation (SD) of 27 or translated in a coefficient of variation (CV) of 12.4%. For the outpatient capacity, the weekly average was 215 patients with an SD of 42 patients (CV of 19.4%). Despite a relatively small standard deviation, the minimum and maximum numbers observed were far apart; the range of weekly demand was 159 to 275 and 124 to 313 for capacity.

#### 3.2.2 Follow‐up consultations

A total of 15 377 requests for follow‐up appointments were registered. A total of 15 318 follow‐up consultations were seen from week 2 until week 46.

The number of follow‐up appointments registered weekly during the year was 334, with an SD of 33 (CV of 10.0%). The capacity, formed by the average number of follow‐up consultations seen per week, was 333, with an SD of 65 (CV of 19.6%). Again, a big fluctuation for both demand and capacity was noticed with a minimum and maximum number of 172 and 462 patients, respectively, for demand and 245 and 380 patients, respectively, for capacity.

When demand and follow‐up consultations were studied in more detail, the data showed that 29% of the registrations of follow‐up consultations were registered directly after previous consultations. In other words, part of the future demand was created directly after supply. Because of this bias, only capacity—or the moment of the actual appointment—was used for further analyses.

#### 3.2.3 Access time

The average access time for all new consultations, measured from the moment of registration until appointment, was 9.8 days, with an SD of 1.6 (CV of 16%). From week 38 onwards, the trend of a rise in average access time is seen (Figure 3). This rise was probably due to queuing because of a mismatch in demand and supply, especially after the summer holiday from week 29 to 34.

### 3.3 Step 3: trends and striking characteristics

#### 3.3.1 New consultations

**Figure 1:**
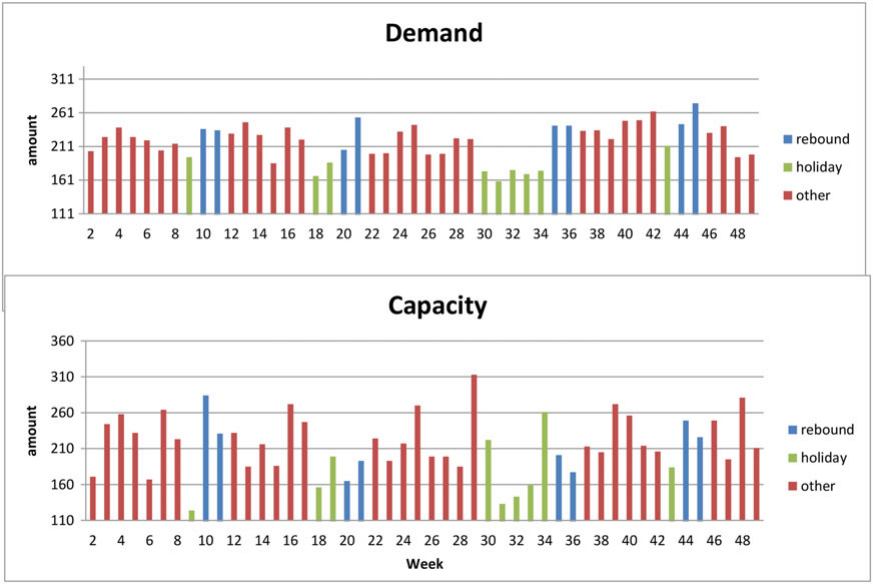
Weekly average demand and capacity

For both demand and capacity, certain weeks seemed to dip notably (Figure 1). These—lower than average—weeks turned out to correspond with holiday weeks. For demand of new consultations, a “rebound” effect was seen directly afterwards in which an above average number of patients was registered. From a regular system perspective, this rebound effect can be described as a temporary rise is demand after holidays due to postponement of demand during holidays. In line with the queuing theory, cumulation of requests for demand during holidays results in a rise afterwards. This probably affects capacity and access time as well as described in the literature.^6^, ^7^, ^16^, ^18^

Although not all peaks and falls were explained with this correspondence, all 9 holidays weeks—divided by 4 separate holidays—were clearly noticed in the graph. The apparent effect of holidays was most clearly seen for demand of new consultations. The average number of requests of consultations during holiday weeks was 178 (SD of 15.7), compared with an overall average of 218. During the 2 weeks afterward holidays, a rise to 241 (SD of 19.3) requests per week was noticed. The same effect during holidays was seen for the capacity of new consultations with a fall to 173 (SD of 43) patients during holidays, compared with an overall weekly average of 215. Remarkably, the rebound effect seen for demand was not seen for the capacity; the average for the 2 weeks after holidays was only 216 (SD of 28.4, CV of 13.2%).

**Figure 2:**
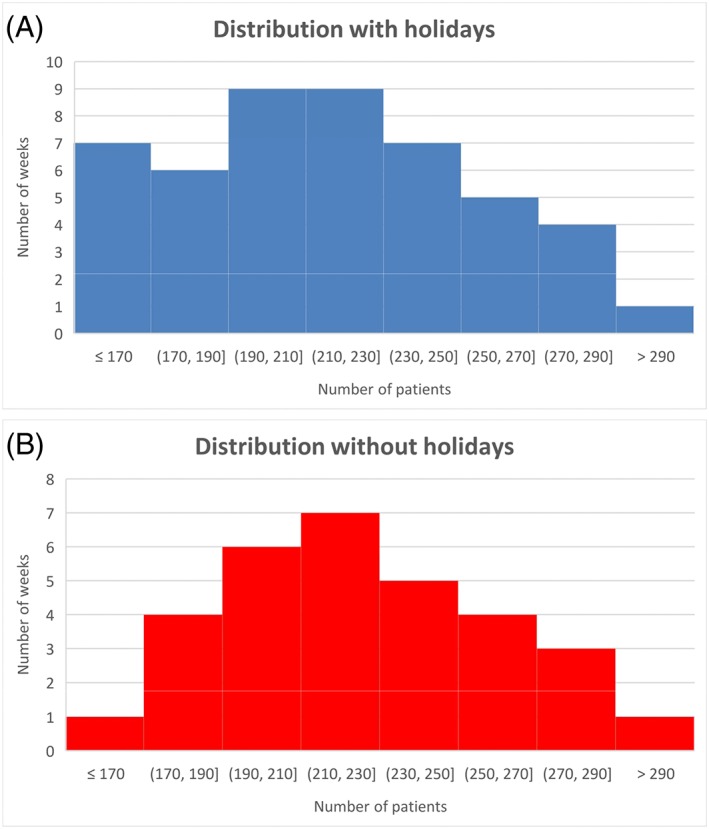
The distribution of demand

These numbers can also be translated to percentages to gain a better general sense of the major effect of holidays on the outpatient logistics. When the demand was translated in percentages, a drop of 20% compared with the overall average was seen during holidays and a rise of 9% was noticed for the 2 weeks afterwards. The same is seen for the distribution of weekly averages; a clearly more compact distribution of weekly averages is seen without holiday and rebound weeks (Figure 2A), compared with the distribution of all analysed weeks. The weekly average without holiday and rebound weeks rose from 215 to 223 requests for new consultations, with a coefficient of variation of respectively 8.6% and 12.4%. When these averages are considered as statistically normally distributed, the effect is clearly seen as well (Figure 2B).

As stated above, no rebound effect was noticed for the capacity of new consultations. Exclusion of only the holiday weeks also resulted for capacity in a more constant average. The weekly average rose to 223 with a coefficient of variation of 8.6%, compared with 215 and 19.4% of all weeks, respectively.

#### 3.3.2 Follow‐up consultations

Follow‐up appointments were also influenced by the holidays. During the year, the weekly number of follow‐up consultations was 333 with a coefficient variation of 24.9%. However, the average number of follow‐up appointments during holiday weeks was only 262 patients (CV of 24.8%), while for the 2 weeks afterwards was 355 with a coefficient variation of 24.9%. Taking the high weekly variation into account, the effect of holidays and consequent rebound weeks was less extreme compared with demand and capacity of new consultations. Assuming a statistical normal distribution, the average capacity during holidays and the 2 weeks afterwards was located at point 13.7% and 63.2% of the normal distribution.

#### 3.3.3 Access time

**Figure 3:**
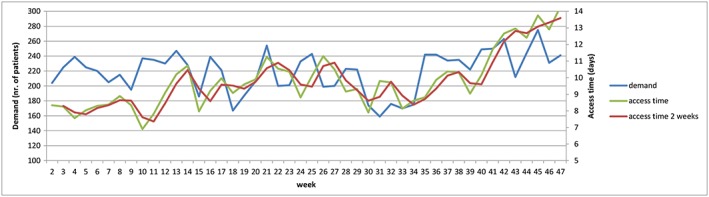
The weekly access time, the fortnightly access time, and demand

**Figure 4:**
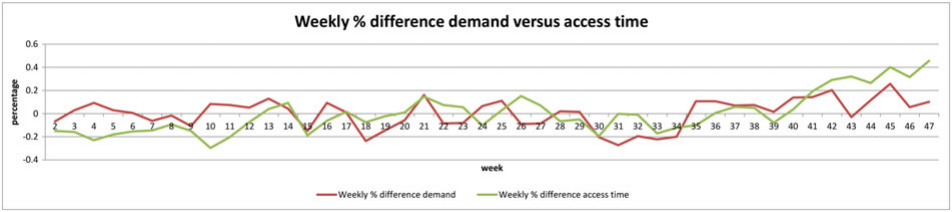
Weekly difference of the access time and demand in percentages

When the graph of the weekly average access time is considered, a big variation is seen (Figure 3). On the first sight, the holiday and rebound weeks do not clearly stand out. When the access time is however depicted by its weekly percentage difference, all holiday weeks and following rebound weeks can be noticed again (Figure 4).

A straightforward hypothesis for outpatient clinics is that when demand rises, the access time rises consequentially. When this correlation between the weekly average demand for new and access time is analysed, a Pearson correlation coefficient of 0.38 is found, indicating that the access time is partially linearly correlated to the demand of new consultations. Another straightforward hypothesis is that the access time falls when capacity increases. The weekly capacity did not however correlate clearly with the access time since the Pearson correlation coefficient was only 0.12.

A more smoothened and probably realistic representation of the access time was seen when the average of every 2 weeks instead of 1 week was used. The Pearson correlation coefficient for this access time in relation to demand was rose using this line with *P* = .43. This was not the case for capacity, with again coefficient of *P* = .12. Both correlation coefficients probably change again when even longer time frames are compared. However, to be able to improve the triad of demand, capacity, and access time, intervals with value in daily practice are important. The average access time (9.8 days) and external factors (such as holidays) correspond with the selected timeframe of 1 to 2 weeks.

#### 3.3.4 Organization of the outpatient clinic

Another factor for improvement might be the organization of the outpatient clinic. Both the number weekly shifts (from 21 to 51 morning and/or afternoon shifts per week) and the ratio between new and follow‐up consultations seen per shift varied greatly (Figure 7). This leads to big differences in workload and—due to more variability in capacity—probably longer access times as well. This hypothesis was underlined by a simple example; during the analysed year, 44% of the weeks showed sufficient capacity to meet the complete demand of that particular week. If the overall average of 5.6 new patients per shift was kept up, this would already have been 54% of the weeks.

Another factor of improvement is the ratio between new and follow‐up consultations of patients seen. The average ratio was 1.59 in favour of follow‐up consultations, with a coefficient variation of 10%. During holidays, this ratio was almost equal with an average of 1.57. However, directly afterward holidays, this ratio raised to 1.66, a remarkable finding, since the opposite was desired. Directly after holidays, relatively more capacity for new consultations was needed because of the rebound effect of demand. This required rise in capacity for new consultations corresponds with a lower ratio contrary to the higher ratio that was actually seen.

## 4 DISCUSSION

### 4.1 Step 4: evaluate demand, capacity, and access time

**Figure 5:**
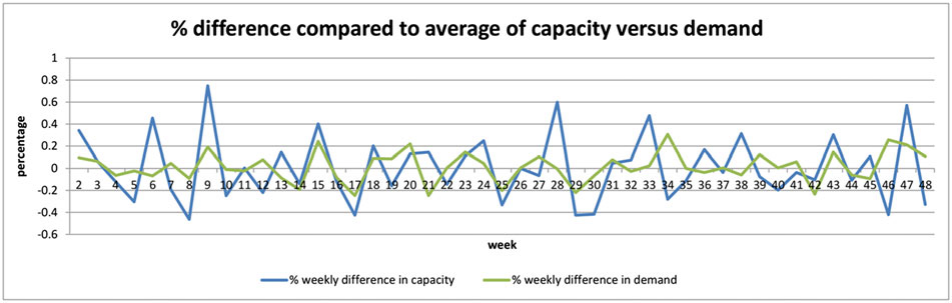
Weekly difference of demand and capacity in percentages

The analysis showed that the weekly variation in demand (CV of 12.4%) was far more constant compared with supply (CV of 19.4%), while the weekly average for demand and capacity was comparable (218 versus 215). This difference can already be noticed when both parameters are simply graphed in weekly percentage differences (Figure 5). These results show that capacity seems a bigger logistical challenge than demand. This is contrary to the sometimes heard preconception of demand being the unpredictable bottleneck in outpatient logistics.

As the results show, the second striking outcome is that holidays turned out to be of big influence of demand, supply, and access time. For demand of new consultations, a postholiday rebound effect was seen, while this was not the case for capacity. This finding suggests that no adjustments in capacity were made in response to the rebound effect seen in demand. With the regular system perspective in mind, it can be expected that a better match between demand and supply favours the access time as well.

In step 3, the influence of holidays for demand was calculated by absolute differences compared with nonholiday weeks. Another way of evaluating the influence of holidays by a time series analysis. In other time series analysis, seasons or months are frequently evaluated. In this analysis, the year is divided by 3 “seasons” or series, which are holiday weeks, rebound weeks, and other weeks.

**Figure 6:**
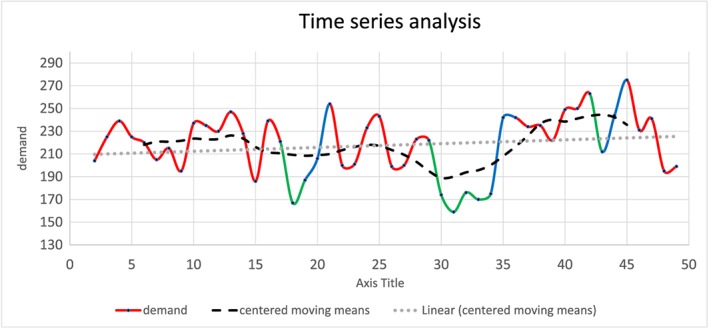
Time series analysis

In Figure 6, the results of a time series analysis are depicted. The graph shows the absolute demand per week, centered moving means of 8 consequent weeks, and a linear trend line. Since this analysis is only depicting 1 year, the results are of limited (prospective) quality. When the average of the holidays was compared with the moving means, an average difference of −9.0% is seen, for rebound weeks was this difference +9.6%, and for the other weeks +1.3%. The difference for the 3 series compared with the trend line was comparable with, respectively, −8.4%, 11.1%, and 3.6%.

**Figure 7:**
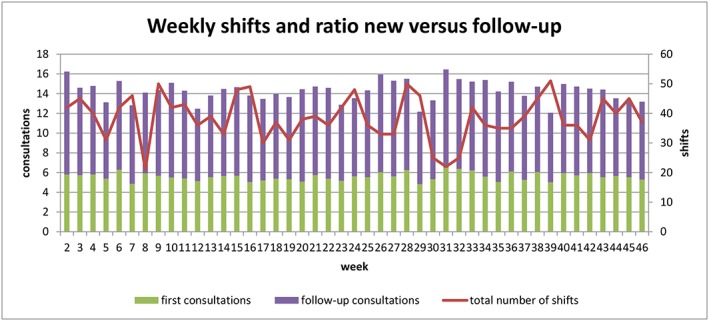
Number of weekly shifts and average weekly ratio

As described in Section 1, the literature frequently focusses on a perfect match between demand and supply.^7^, ^9^ In this clinic, the Pearson correlation coefficient between demand and supply in this clinic was 0.43, indicating a moderate linear correlation. However, when the holidays and rebound weeks were excluded, the Pearson correlation coefficient dropped to only *P* = .23. The same trend was seen for follow‐up consultations, with a correlation coefficient of *P* = .62 with holidays and a decrease to *P* = .42 without. This suggests that the correlation between demand and capacity was mainly due to the influence of the holidays and not because of a direct “match” between demand and supply (Figure 7).

Nevertheless, a fairly stable and highly comparable demand and supply were seen when all holidays and their rebound weeks were excluded. When these weeks were excluded, both demand and supply turned out to have an average of 223 and coefficient variation of 8.6%. This shows that weekly variability (due external factors such as holidays) seems to form a bigger problem that is an absolute mismatch of average demand and supply. This corresponds to the literature in which variability is frequent as one of the main factors leading to delays in access time 9, 12

### 4.2 Step 5: a model for predicting future demand and capacity

In the last research question, the need for “tools” is expressed to support decision making in daily practice. When a blueprint is available to assess and predict outpatient logistics, delays in access time and wastes of capacity can be minimized. A model for forecasting demand, scheduling capacity, and supporting decision forms the last step in the 6‐step model. This model can be applied on the studied outpatient clinic with the data and results of the previous steps as an example. In this example and for this clinic, “only” a single year was analysed. A longer (time series) analysis probably leads to more reliable and therefore usable results. Nevertheless, the steps in this model would be the same.

Before a model for future demand and thereby needed capacity can be created, certain characteristics and limitations of the actual clinics need to be known. The most important characteristics that need to be known are the desirable degree of variation in capacity (in other words, minimum and maximum possible capacity) and the desirable degree of available capacity to meet demand directly. These data are needed be able to do a forecasting analysis.

The model can be demonstrated using the data of the studied outpatient clinic combined with results of the first 4 steps of the 6‐step model. The model can be used to give policy makers a blueprint for holiday, nonholiday, or rebound weeks to estimate the needed capacity for new consultations. Imagine that the studied clinic is designed for a minimal capacity of 37 shifts per week but is able to stretch capacity to 42 shift per week without having to make major logistical changes. In addition, it is decided that the aim must be see new consultations within a week in 90% of the weeks.

As the 6‐step model has showed, the normal distribution in this clinic is an average of 223 new demands per week with a standard variation of 19. The results showed also that the average number of new consultations per shift was 5.6. In this example and for this particular outpatient clinic, it was decided that maximal 6 new patients per shift could be seen, and the minimal and maximum number of shifts per week were 37 and 42, meaning a total of, respectively, 222 and 252 new consultations per week.

**Figure 8:**
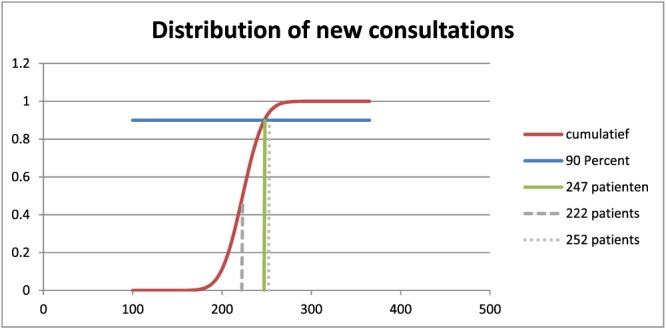
Cumulative normal distribution of demand with the 90% point

With these results, a blueprint for a decision model can be made. As Figure 8 shows, the minimal and maximal capacity for new consultations corresponds with points 0.48 and 0.94 in the graph. This means that using maximum capacity, it is expected that in 94% of the weeks all patients can be seen within a week. On the other side, when the minimum capacity is used, it is only possible in 48% of the weeks to plan new consultations within a week. In this situation, it is expected that during the other 52% of the weeks, no waste of capacity is expected, since (more) than all shifts can be filled in directly. If a more business‐like approach is used, it can be said that with minimal capacity, there is a 52% chance of reaching “break‐even” (no waste of capacity), but with expected delays in access time.

For this clinic, it was decided that for 90% of the weeks, capacity should be sufficient to meet demand. This point corresponds with point 247 in graph 7. This number corresponds the closest with 41 shifts, which is less than maximum capacity and thereby being a realistic policy.

The same can be done for holiday weeks of follow‐up consultations, leading to a more detailed forecast during the year. Of course, for both new and follow‐up consultations, increased capacity on top of the minimal capacity leads to the risk of overcapacity, but less delays in access time, a balance that had to be weighted by the decision makers. To minimalize spill, the individual blueprints could be combined. An option is the usage of flexible shifts in which slots are held free for either new of short‐term follow‐up appointments. For example, in this outpatient clinic, 15% of the follow‐up appointments were registered within the previous 7 days. By setting up shifts that consist of fixed forecasted and flexible parts for either short‐term follow‐up appointments or weeks with a relatively high demand for new consultations, overcapacity might be reduced.

By this way, usage of the 6‐step method might lead to a more efficient triad of demand, capacity, and access time.

## 5 CONCLUSIONS

### 5.1 Step 6: conclusions and potential improvements

The formulated 6‐step model showed to be comprehensive enough to discoverer different trends of the outpatient logistics. The model showed that that not only the specific results of the analysis but also the method itself can be of great value for other clinics.

While the above conclusion answers the first research question, the second research question was focused on finding improvements for demand, capacity, and access time of this and other outpatient clinics. As the first 4 steps of the 6‐step model show, different relevant findings and leads for improvement were found. First, it was showed that a linear correlation or match between demand and supply turned out to be quite low in real practice. This differs from the linear correlation that is sometimes assumed in the literature.^8^, ^12^, ^14^, ^15^, ^16^ Secondly, the analysis showed that capacity was more variable than demand, although the overall average of both was comparable. Big variations in access time were observed as well. These conclusions lead to one of the main points of observation and improvement; not only is capacity more easy adaptable than demand, it is also highly likable that ensuring a less variable capacity leads to a better match between demand and supply and favours the access time as well.

Another important finding was that holidays showed to have a big influence on demand. Strikingly, capacity does not seem to react adequately on this. In the light of a redesigned outpatient system, adjustment of capacity to withstand this rebound effect will probably lead to a better match of demand and supply.

When the capacity was seen in context of weekly shifts and ratio's between new and follow‐up consultations, big variations throughout the year were again seen. Simple plotting of these data gave valuable insights and points for improvement, such as a more aware distribution and ratio between new and follow‐up consultations of adjustments of the ratio for certain periods such as during rebound weeks.

Last, the results of steps 1 to 4 could be used in step 6 to create a model to forecast demand, schedule capacity, and support decision making. While the reliability and usability of the results of this model are correlated with the amount and quality of the data, the model can serve as a guideline for capacity planning in outpatient logistics. This study and the 6‐step model can therefore not only be a reference for future research but also be of value for daily practice of the outpatient clinic.
